# Wearable and Invisible Sensor Design for Eye-Motion Monitoring Based on Ferrofluid and Electromagnetic Sensing Technologies

**DOI:** 10.3390/bioengineering10050514

**Published:** 2023-04-25

**Authors:** Jiawei Tang, Patrick Luk, Yuyang Zhou

**Affiliations:** 1 Electric Power and Devices Group, Cranfield University, Cranfield MK43 0AL, UK; jiawei.tang@cranfield.ac.uk; 2 School of Computing Engineering and Built Environment, Edinburgh Napier University, Edinburgh EH10 5DT, UK; y.zhou@napier.ac.uk

**Keywords:** wearable electronic device, eye-motion monitoring, ferrofluid, electromagnetic sensors

## Abstract

For many human body diseases, treatments in the early stages are more efficient and safer than those in the later stages; therefore, detecting the early symptoms of a disease is crucial. One of the most significant early indicators for diseases is bio-mechanical motion. This paper provides a unique way of monitoring bio-mechanical eye motion based on electromagnetic sensing technology and a ferro-magnetic material, ferrofluid. The proposed monitoring method has the advantages of being inexpensive, non-invasive, sensor-invisible and extremely effective. Most of the medical devices are cumbersome and bulky, which makes them hard to apply for daily monitoring. However, the proposed eye-motion monitoring method is designed based on ferrofluid eye make-up and invisible sensors embedded inside the frame of glasses such that the system is wearable for daily monitoring. In addition, it has no influence on the appearance of the patient, which is beneficial for the mental health of some patients who do not want to attract public attention during treatment. The sensor responses are modelled using finite element simulation models, and wearable sensor systems are created. The designed frame of the glasses is manufactured based on 3-D printing technology. Experiments are conducted to monitor eye bio-mechanical motions, such as the frequency of eye blinking. Both the quick blinking behaviour with an overall frequency of around 1.1 Hz and the slow blinking behaviour with an overall frequency of around 0.4 Hz can be observed through experimentation. Simulations and measurements results show that the proposed sensor design can be employed for bio-mechanical eye-motion monitoring. In addition, the proposed system has the advantages of invisible sensor set-up and will not affect the appearance of the patient, which is not only convenient for the daily life of the patient but also beneficial for mental health.

## 1. Introduction

Early detection, early diagnosis, and early treatment are significant topics in the field of medicine. Medical diagnosis is the process of monitoring and detecting symptoms and medical signals, and an increasing number of people have recognised the significance of regular medical evaluation [1,2,3,4,5,6]. A medical sign is an indicator of a medical condition and is crucial for medical diagnosis. For instance, irregular eye blinking can serve as a diagnostic indicator for dry eye syndrome [7,8,9], burning mouth syndrome [10,11,12], progressive supranuclear palsy [13,14,15], and even autism [16,17,18]. Abnormal motor behaviour, such as tremors and rigidity [19,20], may be a symptom of alcohol intoxication, hypokinesia, hyperthyroidism, hepatic illness, parkinsonism, etc. Since treatment is more effective in the early stages of disease, early diagnosis of these abnormal bio-mechanical behaviours is crucial; hence, wearable and simple detection methods are essential.

Monitoring eye blink frequency is essential to assess the health of human eyes. Blinking plays a crucial role in lubricating and protecting the eyes, and an abnormal blink rate can indicate various conditions that affect visual health. One of the common factors that can lead to a decrease in blink rate is prolonged screen time. Therefore, monitoring blink frequency can be useful in assessing eye tiredness, which is a condition caused by prolonged visual tasks, such as reading, working on a computer, or driving for long hours [21].

The principle of eye tiredness assessment through blink frequency monitoring is based on the fact that blinking helps to maintain eye moisture and prevent dryness. When an individual focuses on a screen for an extended period, they tend to blink less frequently, causing eye dryness and fatigue [22]. Therefore, by monitoring blink frequency, doctors and researchers can better understand the impact of visual tasks on eye health. Furthermore, the analysis of blink frequency patterns over time can reveal trends and changes that may indicate early signs of eye diseases.

In addition to eye tiredness, monitoring blink frequency can also aid in the early detection of potential eye diseases. Studies have shown that a decreased blink rate is associated with dry eye syndrome, a condition that affects the quality and quantity of tears. Dry eye syndrome can cause discomfort, irritation, and even vision loss. By recognising and tracking changes in blink frequency, doctors can provide early intervention and treatment to prevent or mitigate the progression of this disease. Furthermore, monitoring blink frequency can also aid in the early detection of Parkinson’s disease, a neurological disorder that affects movement and balance. Parkinson’s disease is known to affect the activity of the brain regions responsible for eye movement and blinking. As a result, individuals with Parkinson’s disease tend to have a reduced blink rate, which can be a symptom of the disease. By monitoring blink frequency, doctors can detect early signs of Parkinson’s disease and provide timely treatment [23]. In summary, monitoring eye blink frequency is a valuable tool in assessing eye health and detecting potential eye diseases. It can provide insight into eye tiredness, help individuals and healthcare professionals take necessary steps to protect and preserve their visual health, and aid in the early detection of conditions such as dry eye syndrome and Parkinson’s disease. By paying attention to blink frequency, individuals can take proactive measures to maintain good eye health and prevent vision loss.

Certain bio-mechanical or biological signals can be used as a method of communication between humans and electronic devices. Human–machine interfaces (HMIs), a way of communication between a human and an external device that converts a virtual thinking into a real-world action, have attracted increasing interest from researchers [24,25,26]. HMIs that use bio-mechanical/biological signals to express feelings/emotions or operate external devices offer “hands-free” advantages [27,28]. Consequently, HMIs are especially beneficial for people with motor problems. Accurate identification of biomechanical/biological signals serves as the foundation for subsequent phases, such as expression and regulation. Electroencephalogram (EEG) [29], electrooculogram (EOG) [30], electrocorticogram (ECG) [31], and electromyogram (EMG) [32] signals are frequently utilised biomedical signals. Each of these biological signals has its own applications, as well as its own pros and limitations. Generally, ECG is an easy-to-perform, highly informative, and useful diagnostic tool. EEG is a low-cost and easily repeatable technique. However it suffers from low spatial resolution and poor signal-to-noise ratio (SNR). EMG is used to measure the electric potential between muscle cells, but it requires an insertion of needle electrodes into the human body and is susceptible to disturbance [29,30,31,32].

Wearable eye-motion monitoring technologies have been widely studied in recent years. Researchers have explored various sensing technologies such as electrooculography (EOG), electroretinography (ERG), and video-based methods to monitor eye motion [33,34]. However, many existing devices have limitations in terms of practicality and concealment. For example, some devices require physical contact with the eye or are intrusive in similar ways, while others require power input for sensors, making them larger and less convenient. In contrast, the proposed method provides a unique solution to these problems. It uses a non-contact and invisible sensor design that does not affect the appearance and daily life of the patient, and it does not require power input for the sensors, which makes the system smaller than those systems with power input, and no risk of loss of power. The unique features of the proposed method, including invisible sensor design and smaller size system, make it a promising candidate for wearable eye-motion monitoring, especially for daily use in healthcare applications.

In this paper, a novel method for detecting bio-mechanical eye movement is proposed. The proposed method utilises electromagnetic induction sensing techniques and ferrofluid-based eye make-up to detect bio-mechanical eye motions, such as the frequency of eye blinking, which is beneficial for tiredness assessment and other potential eye diseases such as dry eye syndrome (DES) [7,8,9], conjunctivitis (pink eye) [35,36] and keratitis [37,38]. Previous work carried out by Y. Xie has introduced a way of EM sensing monitoring by connecting EM sensors to commonly worn items (such as fake eyelashes and gloves) such that the relative mechanical movement between eyelids and joints can be tracked by observing the electromagnetic signal between the attached EM sensors [39]. However, this method requires a constant signal generation to measure the mutual inductance of the sensors. More importantly, the wearable sensors on the eyelashes are attached with wires and cables which significantly affects the appearance of patients and it might attract public attention in daily life, which is unacceptable for some of the patients. However, this paper proposed a novel method of eye-motion monitoring based on ferrofluid materials and invisible coil designs.

Ferrofluid was discovered by the National Aeronautics and Space Administration (NASA) in 1963. It is a colloidal suspension of ferro-magnetic nano-particles in a liquid medium. In the past few decades, the super-paramagnetism and miniaturisation features of ferrofluids have been the subjects of extensive research and scholarly articles [40]. They have been the subject of substantial study for electromagnetic engineering, mechanical engineering, and medical applications. With the rapid advancement of these cutting-edge technologies, the importance of ferrofluid research and development has grown [41,42,43]. In this paper, ferrofluid is used as a magnetic medium for electromagnetic signal detecting. Due to the black colour and liquid form of the ferrofluid, it can function as an ’eye liner’ on human eyes as a make-up. Then, a fake eyelash with small permanent magnet attached is attracted by the ferrofluid and thus the fake eyelash is adsorbed firmly on the eyes. With the eye motion of blinking, the permanent magnet generates a variable magnetic field, which can generate an electromagnetic induction on a couple of passive coils winding on the glasses. The coils are designed to adapt the shape of the frame of the glasses, which makes the system wearable and the coils are made potentially invisible by integrating them into the frame of the glasses. The energy of the signal is harvested from the eye-blinking motion itself and does not require any external energy for continuous signal generating. Furthermore, each signal generated by the eye-blinking motion can be recorded for the treatment of eye diseases.

The use of ferrofluid as eye make-up may pose potential side effects for extended periods. One such side effect is the difficulty in removing ferrofluid from the eye, which necessitates the use of cleansing agent that might irritate the eye and increase the risk of eye infections. Additionally, the nanoparticles of ferrofluid will not be firmly attached to the skin indefinitely, which means that the patient needs to apply the eye makeup periodically. It might cause inconvenience for the patient to put on and remove the eye make-up regularly. However, the advantages gained from the proposed method are significant enough to justify the potential side effects caused by the ferrofluid as an eye make-up.

In this paper, both quick blinking with an overall frequency of around 1.1 Hz and slow blinking with an overall frequency of around 0.4 Hz can be tracked. A finite-element-based simulation is carried out to simulate the signal generated from the eye-blinking motion.

## 2. Model

### 2.1. Design Model

**Figure 1:**
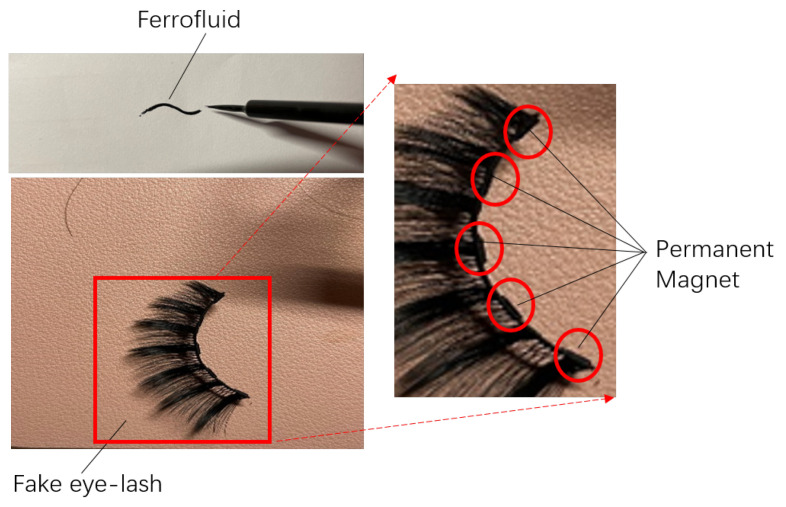
Ferrofluid-based eye-liner and eyelash attached with permanent magnet.

**Figure 2:**
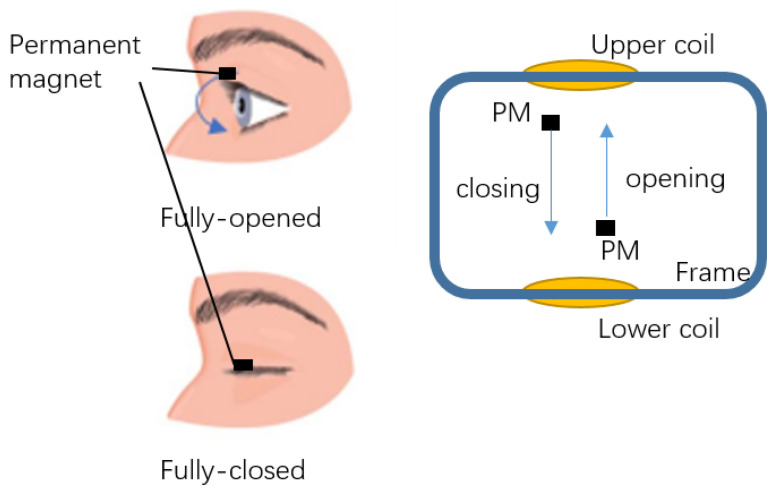
Permanent magnet moving with eye-blinking motion.

In order to apply early treatment of eye diseases and to take care of the mental health of the patient, this work proposed a novel wearable eye-motion monitor with ferrofluid-based eyelash make-up, in which the detecting sensors are integrated inside the frame of glasses so that the monitoring devices are invisible and do not affect the appearance and daily life of the patient. Ferrofluid is a super-paramagnetic material that mainly consists of nano-particles of ${\mathrm{Fe}}_{3}O_{4}$ and water which causes no harm to the human body. The colour of the ferrofluid is mostly dark black which makes it a good replacement for eye-liner. By putting small permanent magnets on the fake eyelashes, as shown in Figure 1, the fake eyelashes are easily attached to the ferrofluid eye-liner due to the super-paramagnetic characteristic of the ferrofluid. With the permanent magnets attached to the eyelashes, each blinking motion of the eyes moves the permanent magnet in the vertical direction, as shown in Figure 2, which leads to the change of magnetic field inside the coils and generates an induced signal on the coils integrated inside the frame of the designed glasses. The signal is detected and recorded to monitor the eye-blinking motion for early treatment of eye diseases.

**Figure 3:**
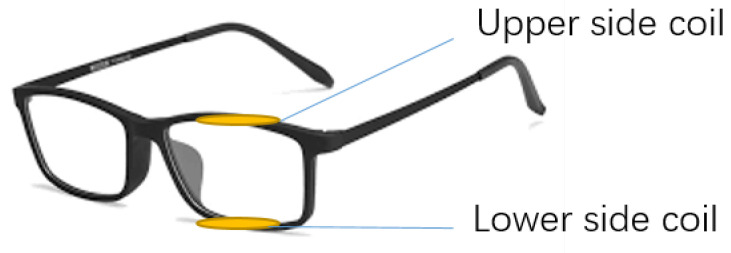
Schematic diagram for designed sensors and glasses.

The model of the designed glasses is shown in Figure 3. The top side and bottom side of the glasses’ frame is hollowed out to put in the sensors to make the monitoring system invisible and avoid influencing the appearance and daily life of the patient. The upper-side coil and lower-side coil are connected in the same direction so that the induced signal can be multiplied into a larger signal which is easier to be detected and more sensitive to the eye-blinking motions. The proposed eye-motion monitoring system is designed to be adaptable to various eye shapes and sizes. The wearable sensor system is embedded inside the frame of the glasses, which can be customised to fit different head and face sizes. Additionally, the system employs a non-contact sensing approach, where the sensor does not directly touch the eye surface, thus reducing the potential for discomfort and ensuring the system’s adaptability for various eye shapes. The use of a ferrofluid-based sensor also allows for flexibility in the sensing area, as the fluid can conform to the shape of the eye. Moreover, the proposed sensor does not rely on external power sources, which means that it can potentially be miniaturised to fit even smaller eye shapes. In this way, the proposed system has the advantage of being adaptable to different individuals’ eye shapes and sizes, which is crucial for ensuring accurate and reliable monitoring results.

This work mainly focuses on the design of an invisible sensing system. The signal-receiving and -recording system is still based on Arduino Mega board. However, both the monitoring system and data-processing system can be potentially integrated into the frame of the glasses in the future work.

### 2.2. 3D Print Model

**Figure 4:**
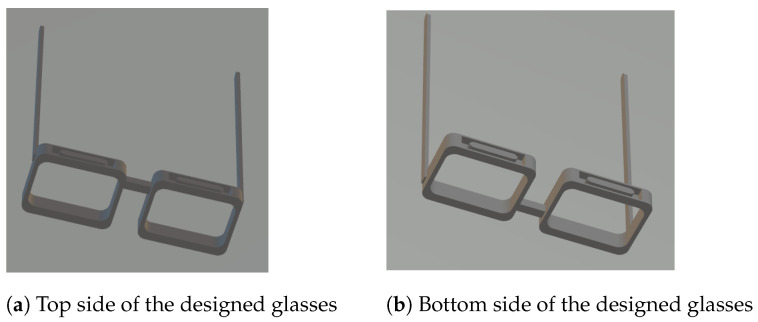
The 3-D printing model for the designed glasses.

To build the specific frame shape of the designed glasses, a three-dimensional (3-D) printing model is built and the glasses are printed using 3-D printing technology. The 3-D printing model is built based on the geometry module of the software Comsol Multiphysics and the file generated is in STL format which is the basic 3-D printing file format. The 3-D printing model is shown in Figure 4. Figure 4a shows the top side view of the model and Figure 4b shows the bottom side view of the model. Both top and bottom side of the frame are hollowed out to put in the coils. The hollowed part is designed as a elliptical ring to fit the shape of the coils. There are columnar parts remaining in the centre of the hollowed part to work as the cores of the coils, which keep the coils in a fixed shape and make it more convenient for coil winding.

### 2.3. FEM Simulation Model

**Figure 5:**
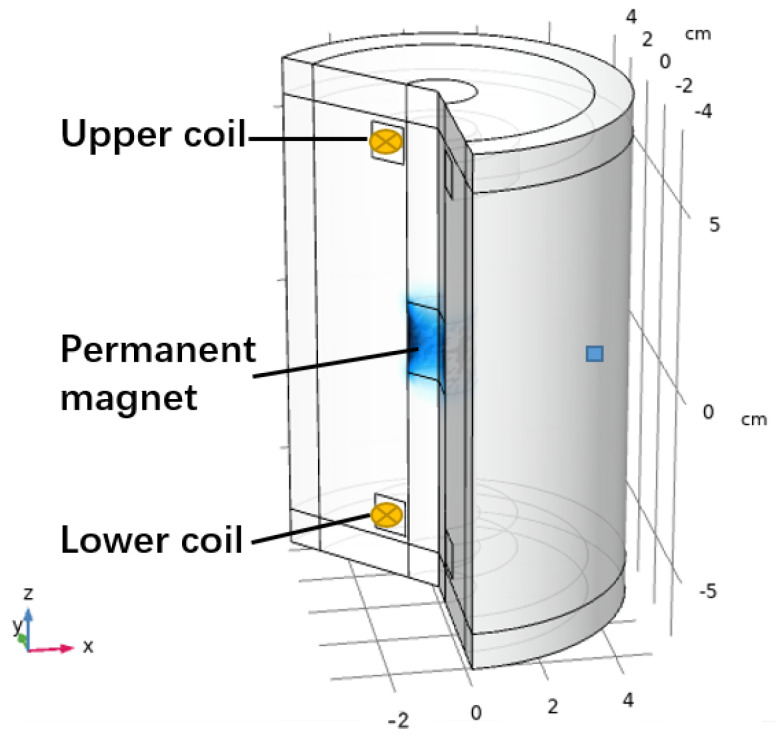
Finite element model for moving permanent magnet generating induced signal in coils.

The finite element simulation model is built based on Comsol Multiphysics to analyse the induced signal inside the coils generated from the moving permanent magnet. The FEM model is shown in Figure 5. The permanent magnet is put in the centre of the model. A dynamic meshing module is used to make the permanent magnet move in simple harmonic motion (SHM), which simulates the permanent magnet movement during the eye-blinking motion. Neodymium is selected as the material for the magnet, and the magnetic properties of the magnet are shown in Table 1.

The coils are put on the top and bottom sides of the permanent magnet, as shown in Figure 5. The number of turns of the coils is set to be 500 and the cross section area of the coil wire is set to be $7.8\times 10^{-3}$${\mathrm{mm}}^{2}$, which is the same size as the wires selected in the experiments. The FEM solver is selected for transient analysis to calculate the induced signal while the permanent magnet is moving vertically. Both upper side coil and lower side coils have the same winding direction and the generated signals inside both coils are multiplied. The moving range of the permanent magnet is set from 0 cm to 3 cm. The work station is based on Windows 10 with 32 G RAM.

## 3. Method

### 3.1. Experimental Set-Up

The frame of the design glasses is built using 3-D printing technology and the material selected is polylactic acid (PLA). The diameter of the wires for winding the coils are 0.1 mm. The number of turns of the coil is 500, which is the same with the finite element simulation model. The data detecting and recording is processed by an Arduino Mega 2560 board. To operate the microcontroller, an Arduino Software (IDE) is built and programmed to read and record the induced signal. In addition, a Python-based program is built to export the data in real time and save the data onto the work station.

**Figure 6:**
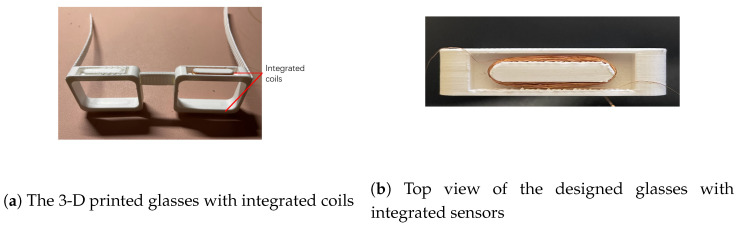
The 3-D printed glasses.

**Figure 7:**
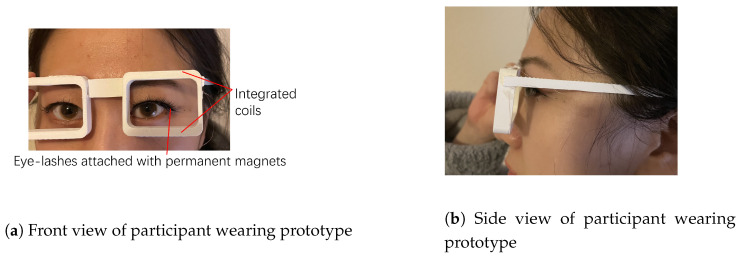
Participant wearing the designed prototype of the glasses with integrated sensors.

The prototype of the designed glasses is shown in Figure 6. The top and bottom sides of the glasses frame are hollowed out for integrating the coils. The diameter of the wire used for winding coils is 0.1 mm. The length of the major axis and the minor axis of the coil are 30 mm and 5 mm, respectively. The thickness and height of the coils are 1 mm and 4 mm, respectively. Figure 6b shows the top view of how the coils are integrated into the frame of the glasses. Figure 7 shows the front view and the side view of participant wearing the glasses. The coils are integrated into the frame of the glasses and they are invisible from the front and the side view. In this work, each end of the coil wires is extended to connect with the signal processor Arduino. However, all the wires can be potentially integrated into the frame of the glasses to make the whole system invisible.

**Figure 8:**
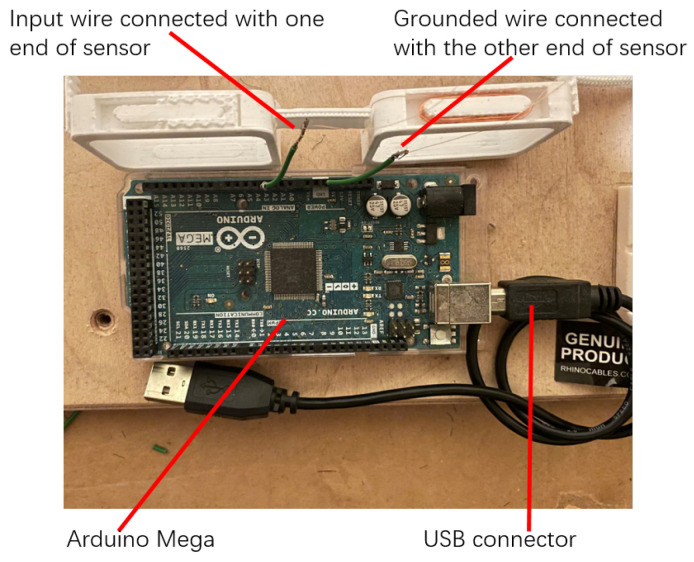
Signal processing system.

The ends of the sensors are connected to the input port and ground port of the Arduino Mega as shown in Figure 8. The signals generated from the sensors are collected by the input port of the Arduino Mega and the measured data are transmitted to the computer by a USB connector for data recording.

### 3.2. FEM Simulation

The finite element model was built to analyse the design of the coils and the induced signal inside the coils while the permanent magnet moves in a simple harmonic motion between the upper and lower coils. Comsol Multiphysics 5.6 is used for building the model, meshing the elements and calculating the induced signals. The governing equation of the magnetic vector potential can be expressed by Equation (1).

(1) $$\begin{array}{c} \nabla \times \left(\mu^{-1}\nabla \times A\right)=j_{c} \end{array}$$

where *A* stands for the potential of the magnetic vector, $j_{c}$ is the density of the current, and μ stands for the magnetic permittivity. The magnetic field of the system *H* and the magnetic induction intensity of the system *B* can be expressed as:

(2) $$\begin{array}{cc} B & =\nabla \times A \end{array}$$

(3) $$\begin{array}{cc} H & =\mu^{-1}B \end{array}$$

The magnetic field of permanent magnet $H{}^{\prime }$ at the no-current region is expressed as:

(4) $$\begin{array}{c} \nabla \times H^{\prime }=0 \end{array}$$

The potential of the magnetic scalar $V_{m}$ is calculated as:

(5) $$\begin{array}{c} H^{\prime }=-\nabla V_{m} \end{array}$$

The intensity of the magnetic induction $B{}^{\prime }$ at the no-current region can be expressed by the following equation:

(6) $$\begin{array}{cc} \; & B^{\prime }=\mu_{0}\left(H^{\prime }+M\right) \end{array}$$

(7) $$\begin{array}{cc} \; & \nabla \cdot B^{\prime }=0 \end{array}$$

where $\mu_{0}$ represents the permeability of vacuum and *M* stands for the magnetization factor. $V_{m}$ can be further expressed by Equation (8), by substituting Equations (5) and (6) into Equation (7).

(8) $$\begin{array}{c} -\nabla \cdot (\mu_{0}\nabla V_{m}-\mu_{0}M)=0 \end{array}$$

The magnetic force of the permanent magnet is applied to the ferrofluid to attract the fake eyelash. The magnetic force can be calculated by integrating the surface stress tensor, which can be expressed as:

(9) $$\begin{array}{c} n_{1}T_{1}=-\frac{1}{2}\left(H\cdot B\right)n_{1}+\left(n_{1}\cdot H\right)B^{T} \end{array}$$

where $T_{1}$ stands for the stress tensor of air, and $n_{1}$ directly represents the normal of the boundary.

According to Faraday’s law, the induced signal can be calculated by:

(10) $$\begin{array}{cc} \varepsilon & =-\frac{d}{dt}\int \int_{S}\vec{B}\cdot d\vec{S} \end{array}$$

(11) $$\begin{array}{c} =-\int \int_{S}\frac{\partial \vec{B}}{\partial t}\cdot d\vec{S} \end{array}$$

The simulation parameters are set according to real-life data, as shown in Table 1.

## 4. Result and Discussion

### 4.1. Simulation Results

**Figure 9:**
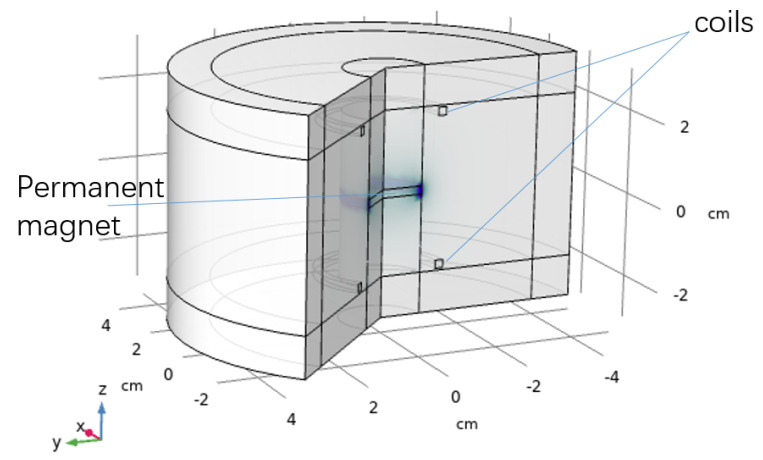
FEM simulation model based on real world data.

The simulation parameters have been set to the same with the real world data, including the size of the permanent magnet and the number of turns of the coils and the position of the magnet and the coils. The revised model based on real world data is built based on the finite element method, as shown in Figure 9. The moving range of the permanent magnet is ranging vertically from 0 cm to 1.5 cm.

**Figure 10:**
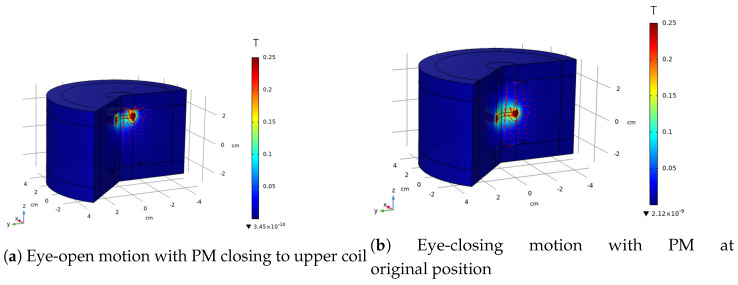
FEM result of eye-opening and -closing position.

The magnetic induction intensity distribution of different permanent magnet positions is shown in Figure 10. Figure 10a shows the simulation result for when the permanent magnet is at position 1.5 cm, which simulates the position of the permanent magnet with the eye fully opened. Figure 10b shows the position of the permanent magnet where the eye is fully closed.

**Figure 11:**
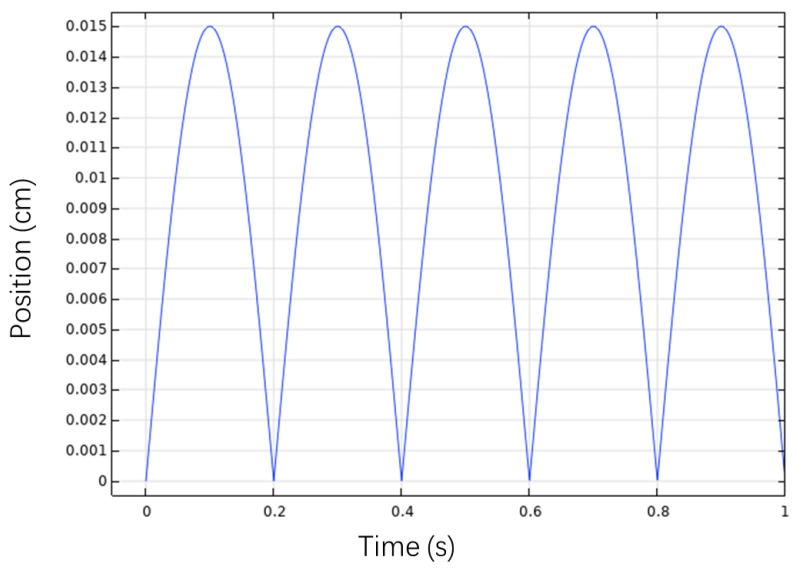
Position of permanent magnet.

The vertical position of the permanent magnet in time domain is shown in Figure 11. A single eye-blinking duration for humans is from 0.2 s to 0.4 s. The magnet’s moving period is set to be 0.2 s to simulate a blink motion. As shown in Figure 11, position 0 cm represents the fully-closed eye motion and position 1.5 cm represents the fully-open eye motion. The permanent magnet moves from 0 to 1.5 cm periodically to simulate the continuous eye-blinking motion.

**Figure 12:**
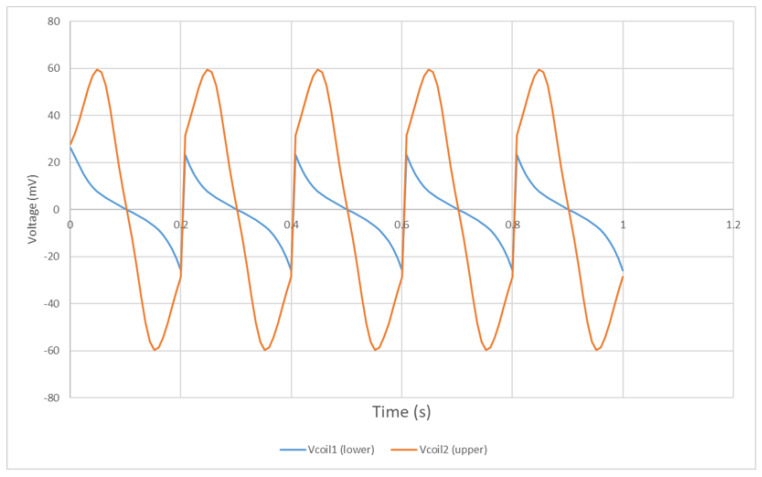
Induced signal inside the coils.

**Figure 13:**
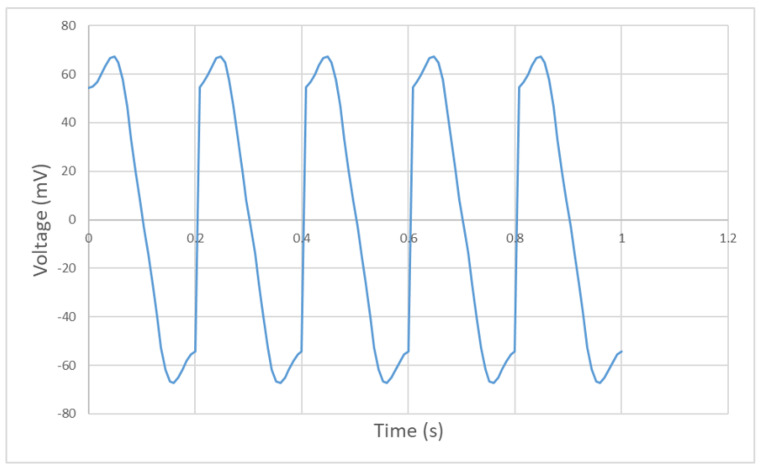
Multiplied signal with a period of 0.2 s.

The induced signals inside the upper and lower coils are shown in Figure 12. The upper coil shows a stronger induced voltage than that of the lower coil, since the permanent magnet is moving on the upper space, which is closer to the upper coil and generates a stronger alternating magnetic field inside the upper coil. As the coils are wound and connected in the same direction, the induced signal can be multiplied into a larger signal for detection. Figure 13 shows the multiplied signal of the upper and lower coils. It generates a positive and a negative pulse during each period of 0.2 s, which can be monitored and recorded to calculate the eye-blinking frequency. Due to the limitation of Arduino, which is used to collect the generated signal, only one positive pulse can be read and recorded in the experimental result. However, it does not affect the monitoring of the eye-blinking motion as each positive pulse detected represents a single eye-blinking motion.

**Figure 14:**
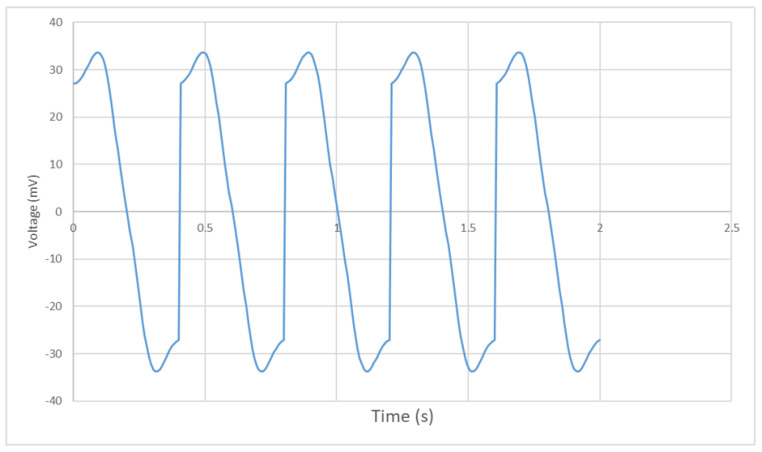
Multiplied signal with a period of 0.4 s.

In order to fully understand the system behaviour for different blinking durations, an additional simulation with 0.4 s blinking duration is carried out. As shown in Figure 14, the multiplied signal still shows a positive and negative peak each period. However, the magnitude of the 0.4 s duration signal is only a half of the 0.2 s duration signal. This is because, when the blinking duration is shorter, the permanent magnet moves faster and leads to a larger magnetic field change in a unit period. Thus, the blinking motion with shorter period generates a larger induced signal inside the coils.

### 4.2. Experimental Results

The designed 3-D printed glasses and integrated sensor for eye-blinking monitoring is shown in Figure 6. The whole system consists of a 3-D printed glasses frame, two 500-turn coils, ferrofluid-based eye-liner and fake eyelashes attached with permanent magnets. The cost of the system is low and all the materials can be purchased easily from local stores or on-line order. With this novel designed system, the eye-blinking motion can be easily monitored without influence on the appearance and daily life of the patient. The diameter of the wire used for winding coils is 0.1 mm. The length of the major axis and the minor axis of the coil are 30 mm and 5 mm respectively. The thickness and height of the coils are 1 mm and 4 mm respectively. Figure 7 presents the appearance of the monitoring system while worn in real life. The coils are invisible as they are integrated inside the frame of the glasses. The ferrofluid-based eye-liner and fake eyelashes attached with permanent magnet are substitutes for daily make-up. With such a design, each eye-blinking motion generates a induced signal inside the coils and the signal is monitored and recorded for early treatment of eye diseases. There is no direct contact between the sensing system and the human body. The only direct contact is the ferrofluid eye-liner, which is harmless to human health. In the light of this wearable design, the eye-blinking motion can be monitored in daily life without any influence on the appearance of the patient.

**Figure 15:**
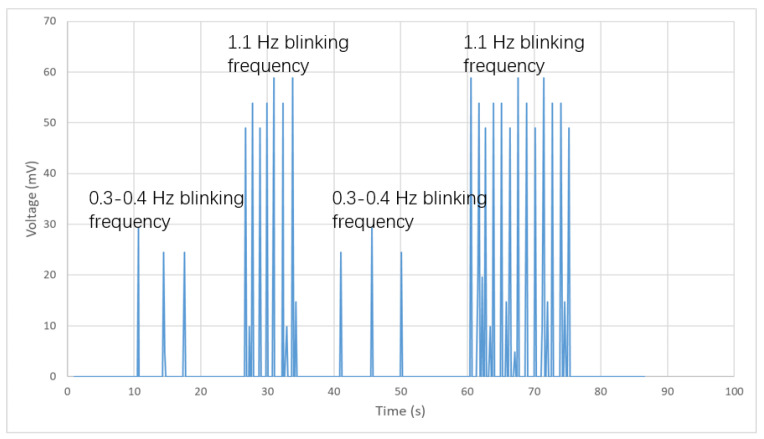
Signal generated and monitored from slow blinking and fast blinking.

The experiment is designed to monitor the eye-blinking frequency. The participant wears the monitoring system and blinks quickly and slowly for a certain period. The measured result is shown in Figure 15. According to the simulation result, each blinking motion generates a positive and negative signal. However, due to the limitation of the Arduino Mega board, only positive signals can be read from the AnologRead port of the board. The AnologRead port of Arduino maps the input voltages between 0 and 5 V into integer values from 0 to 1023, which means the unit for each magnitude of the data is 5/1024V = 4.9 mV. As shown in Figure 15, the participant firstly blinks slowly for 10 s and the frequency measured is approximately 0.3–0.4 Hz. Then the participant stops blinking for 10 s and starts blinking quickly for another 10 s. The frequency measured for fast blinking is around 1.1 Hz. The participant repeats the slow and fast blinking for more data collecting and reduces the system error. Each blinking motion generates a pulse signal and it is used to calculate and record the eye-blinking frequency during the measurement. It is shown in the results that the magnitude of the fast blinking signal is larger than the slow blinking signal, which validates the finite element simulation results. This is because of the fact that, when the participant tries to blink faster, the permanent magnet attached on the fake eyelash moves faster and leads to a larger magnetic field change in a unit time. Thus the fast blinking behaviour generates a stronger signal on the coils than that of the slow blinking behaviour. The proposed wearable monitoring system is working with low cost and high accuracy.

In scientific experiments, it is important to validate the results by replicating real-life scenarios. This is particularly true in the study of eye diseases, where irregular blinking can be a symptom. To imitate this symptom, the participant is asked to blink in a random frequency, alternating between slow and fast blinking. This helps to simulate the unpredictability of the blinking patterns observed in patients with certain eye conditions.

**Figure 16:**
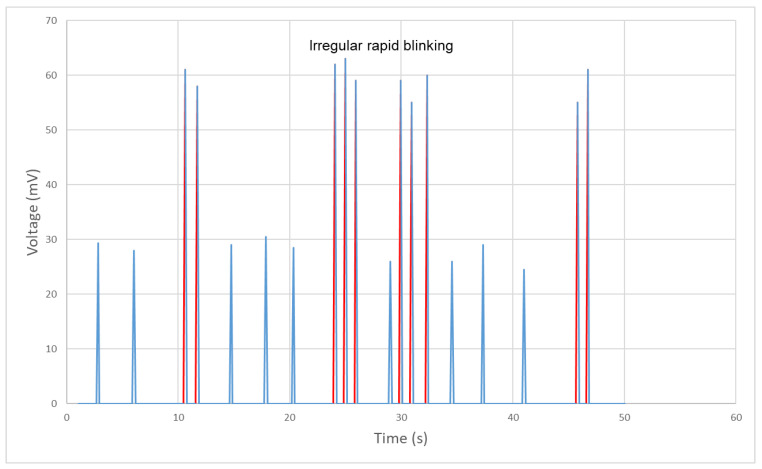
Signal generated and monitored from blinking with random frequency.

As shown in Figure 16, the blue signal represents a normal blinking pattern with a relatively low frequency and consistent speed. This results in a smaller magnitude of the signal compared to the irregular fast blinking pattern shown in red. This fast-blinking pattern is commonly observed in daily life, and can be caused by a variety of eye diseases such as dry eye syndrome, burning mouth syndrome, or even Parkinson’s disease. Due to the higher frequency and speed of the blinking, the magnitude of the signal is much larger than that of the normal blinking pattern, as illustrated in Figure 15. By accurately simulating these irregular blinking patterns, researchers can better understand the effects of different eye conditions and develop more effective treatments.

### 4.3. Discussion

The proposed method for eye-motion monitoring has several advantages over existing systems. One significant advantage is its non-contact and invisible sensor design that does not interfere with the patient’s daily life and appearance. This feature is not present in other monitoring systems that typically require the use of physical sensors, which can be cumbersome and uncomfortable for the patient. Another advantage is that the proposed method does not require any power input for the sensors, making it potentially very small in size. This is not the case for other systems that require power input for sensors, which can increase the size of the monitoring device. Moreover, the proposed method has been demonstrated to be adaptable to various eye shapes and sizes. This is not the case for some existing systems that may have difficulty fitting different eye shapes and sizes, resulting in inaccurate measurements. In terms of accuracy, the proposed method has been shown to be able to accurately monitor eye-blinking frequency, which is a significant indicator of fatigue and some neurological diseases. Furthermore, the proposed method has been shown to be effective in detecting early-stage symptoms of various diseases through bio-mechanical motion monitoring.

Overall, the proposed method offers several unique advantages over existing eye-motion monitoring systems, including its non-contact and invisible sensor design, potential for a small size, adaptability to various eye shapes and sizes, and accuracy in monitoring eye motion and detecting early-stage disease symptoms.

## 5. Conclusions

Diagnosis is a hot topic in both medical research and clinical care practise. Medical diagnosis and treatment are more effective when the biological symptom is detected early. This research examines non-contact sensing for bio-mechanical detection. The design of a wearable monitoring system based on ferrofluid eye-liner and wearable devices makes the system simple to operate and eliminates the requirement for direct contact between electrical sensors and the eyelids. In addition, the devices are invisible, which does not affect the daily life of the patient.

The simulation results are validated with experimental results. The experiment involves detecting the signal generated from eye motions and measuring the frequency of eye blinking. The designed wearable device for monitoring eye blinking is based on a ferrofluid eye-liner, fake eyelashes attached with a permanent magnet, and 3-D printed glasses. The measurement results demonstrate that the quick blinking and slow blinking behaviours can be detected and monitored using the proposed method. Furthermore, the eye-blinking frequency measured in the experiment is identical to that observed in real life. In addition, the amplitude of rapid blinking is greater than that of slow blinking because rapid blinking entails a stronger and faster tissue movement than slow blinking, which generates a stronger induced signal inside the sensors. Future research will concentrate on integrating the whole measurement system into the frame of the glasses to make the whole system invisible and wearable.

## Figures and Tables

**Table 1:** Parameters of Neodymium magnet.

Symbol	Parameter	Value	Units
$B_{r}$	Remanence	1.2	T
$H_{ci}$	Coercivity	1000	kA/m
$BH_{max}$	Maximum energy product	300	$\mathrm{kJ}/m^{3}$
$T_{c}$	Curie temperature	310–400	${}^{\circ }C$

## Data Availability

Not applicable.
